# Intestinal Inflammation and Alterations in the Gut Microbiota in Cystic Fibrosis: A Review of the Current Evidence, Pathophysiology and Future Directions

**DOI:** 10.3390/jcm11030649

**Published:** 2022-01-27

**Authors:** Rachel Y. Tam, Josie M. van Dorst, Isabelle McKay, Michael Coffey, Chee Y. Ooi

**Affiliations:** 1 Discipline of Paediatrics & Child Health, Randwick Clinical Campus, School of Clinical Medicine, UNSW Medicine & Health, University of New South Wales, Sydney, NSW 2031, Australia; z5159530@unsw.edu.au (R.Y.T.); j.vandorst@unsw.edu.au (J.M.v.D.); michael.coffey@unsw.edu.au (M.C.); 2 Wagga Wagga Base Hospital, Wagga Wagga, NSW 2650, Australia; isabellemckay-10@yahoo.com; 3 Department of Gastroenterology, Sydney Children’s Hospital Randwick, Sydney, NSW 2031, Australia

**Keywords:** cystic fibrosis, gastrointestinal tract, intestinal inflammation, gut microbiome, dysbiosis

## Abstract

Cystic fibrosis (CF) is a life-limiting autosomal recessive multisystem disease. While its burden of morbidity and mortality is classically associated with pulmonary disease, CF also profoundly affects the gastrointestinal (GI) tract. Chronic low-grade inflammation and alterations to the gut microbiota are hallmarks of the CF intestine. The etiology of these manifestations is likely multifactorial, resulting from cystic fibrosis transmembrane conductance regulator (CFTR) dysfunction, a high-fat CF diet, and the use of antibiotics. There may also be a bidirectional pathophysiological link between intestinal inflammation and changes to the gut microbiome. Additionally, a growing body of evidence suggests that these GI manifestations may have significant clinical associations with growth and nutrition, quality of life, and respiratory function in CF. As such, the potential utility of GI therapies and long-term GI outcomes are areas of interest in CF. Further research involving microbial modulation and multi-omics techniques may reveal novel insights. This article provides an overview of the current evidence, pathophysiology, and future research and therapeutic considerations pertaining to intestinal inflammation and alterations in the gut microbiota in CF.

## 1. Introduction

Cystic fibrosis (CF) is a life-limiting multisystem autosomal recessive disease most commonly occurring in Caucasian populations [1,2,3,4]. CF is caused by mutations in the cystic fibrosis transmembrane conductance regulator (CFTR) gene, resulting in dysfunction of the CFTR protein. To date, over 2000 mutations have been identified in the Cystic Fibrosis Mutation Database (www.genet.sickkids.on.ca, accessed on 24 November 2021) [5,6,7,8]. While the burden of morbidity and mortality in CF is classically associated with pulmonary disease, CFTR dysfunction also has far-reaching extrapulmonary sequelae [8,9]. These include exocrine pancreatic insufficiency [10], pancreatitis [11,12], liver disease [13,14,15,16], CF-related diabetes [17,18], and male infertility [19,20]. With advances in targeted therapies, the life expectancy of people with CF has increased, but our understanding of the disease is incomplete and there is still no cure [3,4]. Moreover, people with CF now have improved nutritional and pulmonary outcomes and live well into adulthood [21,22,23]. Correspondingly, extrapulmonary complications, including gastrointestinal (GI) issues, have emerged as priorities [24]. In this article, we review two important manifestations of CF in the gastrointestinal tract, namely, intestinal inflammation and alterations in the gut microbiota, with a focus on the existing evidence, pathophysiology, and future research and therapeutic directions of these aspects.

## 2. CFTR in the Gastrointestinal Tract

CFTR is an epithelial cyclic adenosine monophosphate (cAMP)-dependent anion-selective channel [2,7,25]. The CFTR protein is expressed throughout the GI tract, primarily in the small and large intestines. It exists in a gradient of decreasing concentration from the proximal to distal intestine, and its concentration is generally highest in the intestinal crypts [26,27,28]. In the gastrointestinal tract, CFTR is a key player in chloride and bicarbonate secretion, particularly in the duodenum, where pH is critical; this corresponds to the high expression of CFTR in the proximal small intestine [29]. CFTR also indirectly maintains water homeostasis by influencing osmotic pressure through the passage of ions. Additionally, it exerts regulatory effects on other ion channels (i.e., sodium, potassium, calcium, and other chloride channels), maintains tight junctions in the intestinal epithelium, and modulates the pH of secretions [30,31].

## 3. Intestinal Inflammation

### 3.1. The Intestine Is a Site of Inflammation in CF

In light of the homeostatic role of CFTR in the GI tract, there is a robust body of evidence to support the presence of chronic intestinal inflammation in CF. Whole-gut lavage [32], endoscopy [33,34], capsule endoscopy [35,36], and analyses of fecal inflammatory markers [35,36,37,38,39,40,41,42,43,44,45,46,47] have highlighted this phenomenon. Using whole gut lavage, Smyth et al. first reported in 2000 that circulating concentrations of inflammatory markers, including interleukin (IL)-8, IL-1β, albumin, immunoglobulin (Ig) M, IgG, neutrophil elastase and eosinophil cationic protein (ECP), were elevated in people with CF compared to immunologically normal controls [32]. In the same year, Raia et al. reported their findings from duodenal endoscopy and biopsy [33]. They found that although the duodenal mucosa appeared to be morphologically normal in their cohort of 14 patients with CF, the mononuclear cells in the lamina propria of the CF intestine characteristically exhibited an increased expression of immunologic markers such as intercellular adhesion molecule (ICAM)-1, IL-2 receptor, IL-2, interferon (IFN)-γ and cluster of differentiation (CD) 80, which was not observed in non-CF controls with other GI conditions. However, the study only investigated individuals with CF who were at risk of GI disease, including those presenting with recurrent vomiting and raised serum anti-gliadin antibodies (a marker of celiac disease). Subsequently, using capsule endoscopy, Werlin et al. investigated small bowel disease in CF and reported a high prevalence of mucosal pathologies such as edema and ulcerations in patients with CF, even among those with normal fecal inflammatory marker levels [35]. These findings were later echoed by Flass et al., who also utilised capsule endoscopy and reported intestinal mucosal lesions in patients with CF, both in those with and without CF-related liver disease, although the frequency was higher in those with cirrhosis [36]. More recently, Brecelj and colleagues utilised endoscopy to investigate esophageal mucosal integrity and found that compared to children without CF, children with CF exhibited more severe histopathological changes in the esophageal mucosa, even amongst those with CF who did not have gastro-esophageal reflux disease (GERD) [34]. This suggests that there is likely a process intrinsic to CF that is associated with reduced epithelial integrity, irrespective of iatrogenic or pathological interferences. In support of this, CFTR modulation has been shown to promote the resolution of intestinal histopathological changes (i.e., inspissated mucus in intestinal crypts) seen in CF [48].

Elevated fecal inflammatory markers including calprotectin, M2-pyruvate kinase (M2-PK) and rectal nitric oxide (NO) in people with CF have been reported in a relatively large number of studies. Fecal calprotectin has been assessed in the majority of GI-inflammation-related research in CF, and the general consensus is that it is consistently elevated in patients with CF compared to healthy controls [35,36,37,38,39,40,41,42,43,44,45,46,47]. However, the clinical utility of these findings ought to be interpreted with caution because studies regarding fecal calprotectin in people with CF have utilised different reference ranges of calprotectin with great inconsistency in the literature. There is also variability in the performance of commercially available calprotectin assays. Five studies [35,37,38,39,40] conducted from 2004 to 2017 defined elevated calprotectin as a measurement of >50 µg/g, whereas another five [36,41,42,43,44] conducted between 2014 and 2018 used an upper limit of >100 or 120 µg/g. A further three studies [45,46,47] conducted in 2019–2021 defined the upper limit as >200 or 250 µg/g; these studies largely did so on the basis of established cut-off values for the diagnosis and monitoring of pediatric inflammatory bowel disease (IBD) [49,50]. However, prior studies have shown that fecal calprotectin was higher in children with CF compared to healthy controls but lower than in children with IBD [46]. There also appears to be a gradation effect in CF, where fecal calprotectin levels are significantly elevated in pancreatic insufficient (PI) patients but normal or near-normal in those who are pancreatic sufficient (PS) [51]. It remains unclear whether reference ranges that are useful in IBD are equally applicable in CF, especially considering CF-related intestinal inflammation differs clinically from IBD and likely has unique pathophysiology [51]. Furthermore, several studies have reported that pulmonary exacerbations may inflate the levels of calprotectin [52,53,54]. Therefore, the measurement of fecal calprotectin levels in CF requires careful interpretation.

At present, there is no clear consensus on the clinical approach to CF intestinal inflammation. Patients with CF often experience vague, non-specific abdominal symptoms, such as abdominal pain and flatulence, and do not typically present with classic IBD-like symptoms [7,55,56]. The question remains as to whether chronic low-grade intestinal inflammation is simply a natural and benign feature of CF or an under-recognised malady warranting treatment.

### 3.2. Pathogenesis of Intestinal Inflammation in CF

#### 3.2.1. CFTR Dysfunction

**Figure 1:**
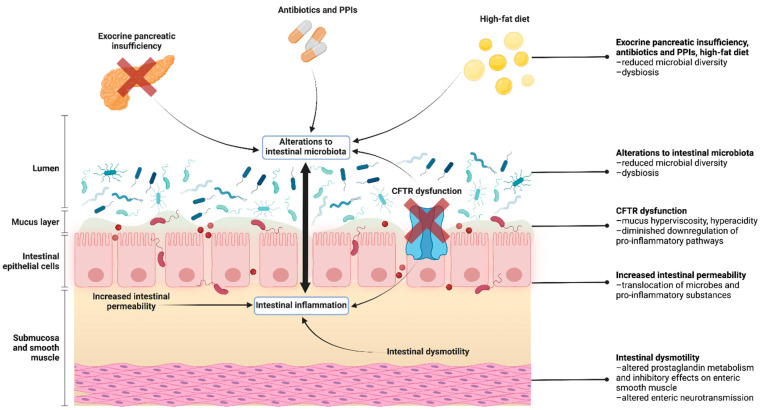
Factors contributing to the cystic fibrosis intestine. The pathogenesis of CF intestinal inflammation and alterations to the intestinal microbiota is multifactorial and complex. A number of intrinsic and iatrogenic mechanisms have been proposed, and it is likely a combination of these mechanisms that culminate in the CF intestine. (This figure was created with BioRender.com.).

The pathogenesis of CF-related intestinal inflammation is likely multifactorial, involving both intrinsic and iatrogenic factors (Figure 1). CFTR dysfunction is postulated to be a major contributor. Faulty ion transport due to CFTR gene mutations leads to mucus hyperviscosity and impaired bicarbonate secretion, which, together, amount to GI dysfunction, hyperacidity, and subsequent inflammation [57,58]. It has also been shown that CFTR itself exerts regulatory effects on inflammatory responses, often by downregulating pro-inflammatory pathways [59]. This is supported by various mouse intestine and human cell line models in which CFTR defects resulted in the upregulation of pro-inflammatory pathways, particularly nuclear factor kappa B (NF-κB)-mediated cascades, which potentiated the secretion of inflammatory cytokines such as IL-6 and IL-8 [31,60,61]. Than et al. also demonstrated that CFTR-deficient tissues exhibit the upregulated expression of pro-inflammatory genes, including the inflammation and oncogenesis-associated S100A gene family [62]. Furthermore, it has been reported that the induction of human β-defensin 2, an anti-microbial protein normally prominent in inflamed states, is impaired in CF even when fecal calprotectin is elevated. This suggests that CF may be associated with a defective enteric innate immune response, which could contribute to an inadequate host response to pathogens and the subsequent development of inflammation [63]. Altogether, these findings are clinically corroborated by recent reports that treatment with CFTR modulators such as Ivacaftor and Lumacaftor/Ivacaftor reduced the level of fecal inflammatory markers in patients with CF, although there have been limited studies [64,65,66].

#### 3.2.2. Intestinal Dysmotility

Intestinal dysmotility and the pooling of inspissated intraluminal contents may be another pathogenetic factor in CF intestinal inflammation and disease [58,67,68]. In animal models, CF has been associated with a prolonged intestinal transit time and enteric muscular dysfunction [69,70,71]. In human studies, Hedsund et al. utilised a radio-opaque marker and reported a significantly increased orocecal transit time in patients with CF compared to healthy controls [72]. Interestingly, however, they noted that the overall contractility patterns and frequencies were similar between both groups, although CF patients demonstrated a normal or increased upper GI transit time and decreased lower GI transit time [72]. Using capsule endoscopy, Malagelada and colleagues also discovered that intestinal contractility was significantly reduced in association with the increased retention of luminal contents in people with CF compared to healthy controls [67]. More recently, Ng et al. demonstrated through novel magnetic resonance imaging (MRI) techniques that orocecal transit times were increased in CF in addition to evidence of increased colonic volumes [73]. The mechanism by which CFTR dysfunction relates to gut dysmotility is unknown, but it has been hypothesised that CF may be linked to alterations in eicosanoid metabolism, resulting in increased levels of prostaglandin E2 that may exert inhibitory effects on enteric smooth muscle [74]. Evidence has also emerged to indicate that CFTR may have critical functions beyond ion transport. It has been demonstrated that CFTR is present in myenteric ganglia and may modulate enteric neurotransmission by mediating acetylcholine release, thereby regulating gut motility [75,76]. As such, dysmotility in CF and subsequent inflammation may, in part, be due to an aberrant enteric nervous system; however, this hypothesis has not yet been extensively explored. 

#### 3.2.3. Intestinal Dysbiosis

Intestinal dysbiosis (i.e., alterations in microorganism composition of the gut) is also thought to contribute to intestinal inflammation. A reduction of commensal bacteria known to have anti-inflammatory properties, such as the *Ruminococcaceae* family, has been observed in patients with CF [77]. These reductions resemble findings in IBD, which suggests that dysbiosis is involved in enteric inflammatory processes [46]. In contrast, pathogenic and inflammation-associated organisms have been documented to be increased in CF. For example, Hoffman et al. reported a significantly increased abundance of *Escherichia coli*, an organism associated with IBD and GI inflammation [78]. This increase in *E. coli* was correlated with fecal calprotectin [78]. Several clinical trials have also identified an association between probiotic administration and decreased levels of inflammatory markers, which may indicate a reduction in, or even reversal of, gut inflammation upon the restoration of healthy microbiota [37,41,79,80]. These findings have been corroborated by meta-analysis [81]. Nevertheless, the physiological link between the gut microbiome and intestinal inflammation remains incompletely understood. More robust and longitudinal trials are needed to validate existing findings in probiotic administration, along with mechanistic studies designed to interrogate the host–microbiome interactions that drive inflammation in CF and vice versa.

#### 3.2.4. Increased Intestinal Permeability

Increased intestinal permeability has been reported in CF, but its relevance to inflammation has not yet been elucidated. Dysfunction of the intestinal epithelial barrier and alterations to tight junctions are thought to undermine epithelial integrity, thus allowing the translocation of microbes and pro-inflammatory substances. This subsequently induces inflammatory changes, which may, in turn, alter the microbiome and promote dysbiosis [82]. In CF mouse models, impaired epithelial function and altered localisation of tight junction proteins have been observed [83,84]. Various human studies using sugar absorption analyses (i.e., urinary lactulose to mannitol ratio) have also shown that intestinal permeability is increased in people with CF [36,85,86,87,88]. Factors reportedly associated with increased intestinal permeability include the delta F508 mutation [85] and pancreatic insufficiency [86,87]. Interestingly, however, Flass et al. found no significant association between increased intestinal permeability and elevated fecal calprotectin [36]. This stands in contrast to multiple studies that have reported a correlation between increased intestinal permeability and IBD [89,90,91]. Nonetheless, it must be noted that epithelial barrier dysfunction alone may not necessarily lead to mucosal inflammation as the complex interactions between the epithelium, immune cytokines, microbiota, and homeostatic pathways also exert a crucial influence on mucosal integrity and health [92]. Further research is needed to examine the relationship between intestinal permeability and GI inflammation in CF, particularly owing to a lack of recent CF-specific studies in this domain. 

### 3.3. Clinical Correlations with Intestinal Inflammation

#### 3.3.1. Exocrine Pancreatic Status, Age, and Lung Function

GI inflammation in CF is not only challenging to understand in itself but is also complicated by dynamic interactions with intrinsic and extrinsic factors that may be of clinical significance. Firstly, the relationship between exocrine pancreatic function and intestinal inflammation remains contentious. Some studies have reported that patients with exocrine pancreatic insufficiency have significantly higher fecal calprotectin than their pancreatic sufficient counterparts [40,42], whereas other studies have found no difference between the two groups [38,53]. Perhaps these conflicting findings reflect the continuum of exocrine pancreatic function seen in the CF population, wherein various mutations and their consequent severities of CFTR dysfunction result in a diverse range of intestinal phenotypes. Furthermore, it is unclear to what extent age relates to gut inflammation. Wiecek et al. [53] reported that elevated calprotectin was more frequent in older children (age > 6 years) than in younger children under 6 years; similarly, Parisi et al. [42] reported that patients over 18 years of age had significantly higher fecal calprotectin levels than patients under 18. Rumman et al. [38] also found that calprotectin correlated positively with age. Interestingly, in a longitudinal study, Garg et al. demonstrated that children with CF paradoxically had lower calprotectin than healthy infants from birth up to 1 year but demonstrated an upward trajectory in fecal calprotectin until the fourth year of life, upon which it remained consistently elevated compared to healthy children [93]. In contrast, fecal M2-PK was consistently elevated in children with CF compared to healthy controls, with no age variation from birth to 10 years [94]. The clinical significance of these conflicting findings is unclear as calprotectin and M2-PK may not necessarily be well-correlated, and M2-PK may reflect increased cellular proliferation and turnover independent of inflammation [94]. The relationship between lung function and fecal calprotectin levels also remains unclear. Some studies [40,46,51] found no association between forced expiratory volume in one second (FEV1) and fecal calprotectin, whereas others [39,42] reported that patients with lower FEV1 had significantly higher fecal calprotectin than patients with better lung function. 

#### 3.3.2. Growth Parameters

Associations between intestinal inflammation and weight and height, both measures of nutritional status, have been reported. Fecal calprotectin has been shown to inversely correlate with height and weight z-scores in children with CF [46,51]. In a mixed cohort of both adults and children with CF, it was also found that elevated fecal calprotectin was associated with underweight status (Body Mass Index < 18.5 kg/m^2^) across all age groups [42]. The direction of the relationship between intestinal inflammation and growth parameters is unclear, but it has been hypothesised that intestinal inflammation may exacerbate the poor growth and malabsorption initiated by exocrine pancreatic insufficiency and CFTR dysfunction [51]. Indeed, treatment with Ivacaftor, a CFTR potentiator, has been correlated with a reduction in fecal calprotectin and weight gain [65,66,95]. These findings provide some evidence to support the exploration of treating intestinal inflammation in order to optimise growth and nutrition. They also highlight the potential role of assessing intestinal inflammation in the evaluation of the efficacy of CFTR modulator therapies.

#### 3.3.3. Quality of Life and Hospitalisations

To date, only one study has directly investigated the relationship between intestinal inflammatory markers and quality of life in CF. Beaufils et al. [47] recently reported that increased fecal calprotectin was associated with worse GI symptoms and quality of life in children with CF. Furthermore, they found that worse GI symptomatology was associated with poorer quality of life. In particular, children with higher fecal calprotectin reported significantly worse emotional functioning, social functioning, and overall quality of life. These findings highlight the possible impact of intestinal inflammation on gastrointestinal symptomatology and, therefore, wellbeing [47]. In another recent study, Sathe et al. [45] found that elevated fecal calprotectin was a predictive factor of GI-related hospital admissions in the first year of life for infants with CF. Failure to thrive or poor feeding was the most common indication for these GI-related admissions, but some of the other reasons included reflux, constipation, and feeding tube placement. In that study, infants with CF who had been hospitalised for GI-related indications also exhibited lower growth parameters than those who had not been hospitalised. Overall, these findings suggest that intestinal inflammation may not only relate to growth but may also have associations with morbidity and wellbeing due to hospitalisations in infancy [45].

#### 3.3.4. Iatrogenic Factors: High-Fat Diet and Antibiotic Use

Due to increased energy expenditure secondary to pulmonary disease and nutrient malabsorption, a high-fat, high-calorie diet has been the traditional nutritional approach in CF to minimise undernutrition [96]. However, contemporary evidence demonstrates that these energy requirements are now more likely to be met via the consumption of saturated fats and energy-dense but nutrient-poor foods [97,98,99,100,101,102]. High-fat diets have been associated with intestinal inflammation in CF as well as in other disease contexts [103,104,105,106,107]. Gulhane et al. demonstrated that long-term high-fat diets, especially those with a high saturated fat content, resulted in low-grade chronic intestinal inflammation in mice by increasing endoplasmic reticulum stress and oxidative stress, inducing inflammatory cytokines and decreasing epithelial barrier integrity secondary to goblet cell dysfunction [105]. While it is difficult to define a clear relationship between diet and gut inflammation independent of the myriad of other intrinsic and iatrogenic factors in CF, the overconsumption of fats, particularly saturated fats, may be one amongst several factors that promote gut inflammation. Hence, diet optimisation (i.e., consuming more monounsaturated fats instead of saturated fats) may be a potential intervention to ameliorate inflammation and improve overall health. 

Antibiotic use is another pertinent iatrogenic factor in CF that is associated with gut inflammation. Using mice models, Knoop et al. illustrated that the administration of oral antibiotics led to an increase in inflammatory cytokines, including IL-17, IFN-γ, and chemokine C-X-C motif ligand 1 (CXCL1). This occurred in conjunction with alterations in gut microbial composition and the translocation of commensal organisms through the epithelium via goblet cell-associated pathways [108]. However, the connection is less clear in human studies. De Freitas et al. did not report any significant differences in fecal calprotectin levels between patients with CF who were on antibiotics at the time of the study and those who were not [44]. Evidently, the influence of antibiotics on gut inflammation remains contentious and requires further investigations. 

## 4. The CF Gut Microbiome

The gut microbiome is a dynamic enteric environment comprised of numerous diverse microorganisms. It confers many vital and complex functions, including the anaerobic fermentation of indigestible nutrients, maintenance of the gastrointestinal epithelium, production of amino acids and essential vitamins, protection against pathogens, and regulation of the immune system [109,110,111]. Its early development is greatly shaped by factors such as one’s mode of birth, diet, and antibiotic exposure [112,113,114]. From early life, the gut microbiome of children with CF exhibits dysbiosis, decreased species diversity, delayed maturation, and altered functionality compared to that of non-CF children. These factors are associated with ill health [77,115,116,117,118]. The intestinal microbiome is comprised of bacteria, viruses, and fungi, all of which contribute to the enteric environment; for the purposes of this review, the focus will be on bacteria, which are by and large the most well-defined constituents. While the roles of the intestinal virome and mycobiome in CF remain largely unexplored, their impact on health and disease, in general, are covered elsewhere [119,120,121].

### 4.1. Species Diversity and Microbiome Maturation

Microbial diversity is a broad term that encompasses the richness (number of species) and/or evenness (abundance of species relative to each other) of an ecological environment [122]. In recent years, reduced microbial diversity has been shown to correlate with a myriad of chronic conditions, including IBD, coeliac disease, obesity, and type 2 diabetes mellitus. It is postulated that a more diverse gut microbiome has a greater capacity to remain resilient against environmental insults and maintain health due to functional redundancy, whereby various species perform similar functions and compensate for one another [123,124]. Reduced species diversity is a hallmark of the CF gut microbiome that is evident in early life and continues throughout adulthood [23,43,77,116,117,125]. While the microbiota of healthy children sees significant increases in diversity with age, the microbiota of children with CF diversifies at a substantially slower rate with each year of life. In fact, it has been shown that even at 15 years of age, the richness of the CF microbiome is unmatched with that of a healthy one-year-old child [117]. Furthermore, microbiota maturation (i.e., the rate of microbiota development) is reduced in CF compared to age-related healthy individuals [115]. Reduced microbial diversity and delayed maturation rates in CF may be reflective of CFTR-related dysfunction or physiological disruptions such as antibiotic use. Reduced diversity is broadly associated with reduced colonisation resistance, mucin production, and intestinal permeability, but the specific implications in CF remain unclear [113,126].

### 4.2. Microbial Composition and Functionality

The advancement of molecular techniques such as next-generation sequencing and multi-omics methods has brought greater insights into the taxonomy of the CF gut microbiota in recent years. Compared to the healthy gut, the CF gut exhibits a relative depletion of the family *Ruminococcaceae* (phylum Firmicutes) [23,43,77,117] and the genera *Bifidobacterium* (phylum Actinobacteria) [43,111,125,127], *Bacteroides* (phylum Bacteroidetes) [46,111,128], *Roseburia* (phylum Firmicutes) [43,116,125,128], and *Faecalibacterium* (phylum Firmicutes) [43,44,46,116,129]. These organisms are all generally considered to be constituents of a healthy gut microbiome as they perform vital functions, including the synthesis of anti-inflammatory metabolites (i.e., butyrate), fermentative processes, protection against enteric pathogens, and immune signaling [43,117,125,128]. Conversely, the CF gut possesses increased abundances of the genera *Enterococcus* (phylum Firmicutes) [77,116,117,125,127], *Enterobacter* (phylum Proteobacteria) [77,111,116,125,129], and *Escherichia* (phylum Proteobacteria) [44,78,127,130]. Notably, an increased abundance of the pathogenic species *E. coli* (genus *Escherichia*, phylum Proteobacteria) is associated with fecal markers of intestinal inflammation and nutrient malabsorption in CF [78]. 

While knowledge of the taxonomic aspects of CF gut dysbiosis has grown, it is perhaps the functionality of the organisms, and not solely their taxonomy, that sheds light on their physiological significance. To this end, knowledge is still lacking, although the homeostatic functions and pathogenic potentials of the microbial communities involved in this dysbiosis are becoming increasingly evident with the emergence of functional data. Coffey et al. reported that the predicted functional profiles of the pediatric CF gut microbiota were significantly different to those of the non-CF gut [77]. In particular, they found that the pediatric CF gut expressed a greater propensity to metabolise short-chain fatty acids (SCFAs), nutrients, and antioxidants [77]. In another study, Manor et al. likewise identified an enrichment of SCFA metabolic pathways as well as a depletion of fatty acid biosynthesis pathways [131]. Interestingly, Wang et al. reported that despite significant dysbiosis, the CF gut microbiota retains the functional capacity to produce SCFAs by mediating the fermentation of starches [132]. Additionally, Matamouros et al. demonstrated that *E. coli* isolates from children with CF exhibited increased growth rates on glycerol, a major constituent of fecal fat [130]. Gene expression in *E. coli* isolates from children with CF and healthy children also differed when grown in glycerol, suggesting that gut microbes may acquire growth traits to adapt to the altered intestinal environment in CF [130]. In essence, it is clear that the altered composition of microbes in the CF gut results in changes to the functionality of the microbiome, but the clinical and therapeutic consequences are still largely unknown.

### 4.3. Pathogenesis of Intestinal Dysbiosis

#### 4.3.1. CFTR Dysfunction

Despite active research, the mechanisms through which CF culminates in intestinal dysbiosis remain incompletely understood. It is postulated that CFTR dysfunction itself is the key driver of dysbiosis. Meeker et al. demonstrated using a mouse model that CF-positive mice exhibited an altered microbiome compared to control mice despite receiving the same donor microbiota, suggesting that CFTR mutations alone drive the selection of bacterial communities in the gut [133]. Furthermore, CF genotypes, which reflect varying classes of CFTR dysfunction, have been shown to affect the extent of dysbiosis. Schippa et al. found that patients with the homozygous delta F508 genotype (the most common and severe CF mutation) exhibited more marked dysbiosis compared to patients with other mutations [134]. Specifically, homozygous delta F508 patients had more significant abundances of *E. coli* and a more marked depletion of *F. prausnitzii* and *Bifidobacterium* compared to other CF genotypes [134]. Interestingly, it has been reported that it may not be the class of CFTR mutation that determines the extent of dysbiosis, but rather the severity of the genotype, that exerts a significant effect on microbial composition [125]. However, another study [43] did not report any significant differences in the gut microbiota between patients with different CF genotypes. The proposed mechanisms by which CFTR dysfunction results in dysbiosis include the production of thick and inspissated mucus, altered intestinal pH due to inadequate bicarbonate buffering, slowed intestinal transit, nutrient malabsorption, and disrupted enteric innate immune responses, all of which exert selective pressure on gut microbes [63,77,78,122].

#### 4.3.2. Exocrine Pancreatic Status

The exocrine pancreas orchestrates fat absorption, and its dysfunction, which results in fat malabsorption, may affect the selection of the gut microbiota. It is hypothesised that certain organisms can adapt to, and eventually thrive in, high-fat intestinal environments, such as that seen in exocrine pancreatic insufficiency in CF [130]. In addition, in healthy non-CF states, the pancreatic ductal epithelium secretes large volumes (1–2 L/day) of bicarbonate-rich alkaline fluid [12]. This pancreatic secretion is physiologically intended to flush digestive enzymes secreted by pancreatic acinar cells down the pancreatic–biliary tree and into the duodenum. In contrast, the pancreatic secretions in CF have lower pH and fluid volumes. Consequently, the downstream small intestinal pH is abnormally lower in CF compared to non-CF states [7]. Nevertheless, the literature reveals conflicting findings on the significance of exocrine pancreatic function in intestinal dysbiosis. Burke et al. [125] and Vernocchi et al. [116] reported no difference in species diversity or taxa between PS and PI patients with CF, whereas Nielsen et al. [117] found that PS patients had higher microbial diversity than PI patients. However, the true effects of pancreatic function on gut microbiota may be masked by the administration of pancreatic enzyme replacement therapy (PERT), and it has indeed been shown in animal models that PERT can restore the diversity and composition of the microbiome [135]. Furthermore, studies are limited in power due to a significantly smaller number of PS patients in the general CF cohort. To complicate and confound, exocrine pancreatic status (PI or PS) is also highly correlated with the degree of CFTR dysfunction in the affected individual. 

#### 4.3.3. Antibiotic and Proton Pump Inhibitor Use

As with intestinal inflammation, the CF gut microbiome is heavily impacted by iatrogenic factors that may be additional contributors to dysbiosis. Antibiotic use, which is prevalent in CF, has been shown to generally alter the gut microbiome both in the short and long term [136,137,138,139,140,141]. Specifically, in the context of CF, antibiotic use has consistently been correlated with decreased alpha diversity (within-sample species diversity) in the gut [115,116,125,127,142]. Moreover, Burke et al. [125] reported that individuals who had received the highest relative number of courses of intravenous antibiotics exhibited the lowest proportion of Bacteroidetes and the greatest abundance of Firmicutes and Veillonellaceae amongst all adults with CF in their study. In a pediatric population, Bruzzese et al. [41] also found that children who were on antibiotics at the time of the study exhibited more marked dysbiosis and had a significant reduction in *Bacteroides* and *Eubacterium rectale* compared to children with CF who had not received antibiotics for at least two weeks prior. Other studies have reported significant associations between antibiotic exposure and a reduction of Bifidobacterium [44,127,142,143]. The CF fecal microbiota may also have a higher prevalence of amoxicillin-resistant *Enterobacteriaceae* due to increased exposure to amoxicillin–clavulanic acid therapy [144]. Additionally, recent studies have highlighted that proton pump inhibitor (PPI) exposure is associated with decreased species diversity and an over-representation of oral and upper gastrointestinal organisms in the gut microbiome. PPI use has broadly been shown to correlate with an increased abundance of *E. coli*, *Enterococcus* spp. and *Streptococcus* [145,146,147]. At present, no clear correlation has been found between PPI use and the gut microbiome in CF, but knowledge is sparse and further research in this domain may provide novel insights [115,125].

#### 4.3.4. High-Fat Diet

Diet plays an important role in altering the gut microbiome [148,149,150]. In mouse models involving a high-fat diet, an increased presence of Firmicutes [151,152,153] and Proteobacteria [153], reduction of Bacteroidetes [106,151,152], and enrichment of *E. coli* [103,152] has been observed. The mechanisms by which a high-fat diet alters the intestinal microbiota are unknown. It is hypothesised that high-fat diets may promote the translocation of certain bacterial communities by increasing intestinal permeability as well as enhancing the abundance of bacterial species that produce lipopolysaccharides [154]. Nonetheless, current knowledge of the impacts of high-fat diets on the intestinal microbiome is dominated by studies on obesity and metabolic syndrome, which will inevitably encompass confounders that are not necessarily reflective of CF.

### 4.4. Clinical Significance of the Gut Microbiome in CF

#### 4.4.1. Pulmonary Function and the Gut–Lung Axis

In recent years, accumulating evidence has highlighted the importance of the gut–lung axis, wherein the intestinal and respiratory microbiota engage in crosstalk and regulate immune responses and homeostasis distally along this axis. Of the two compartments, the far-reaching effects of the gut microbiome have been better characterised [155]. Intestinal bacterial metabolites, primarily SCFAs, orchestrate immune cell signalling cascades that reach the airways through G protein-coupled receptor (GPCR)-mediated pathways and histone deacetylase inhibition [156,157,158]. There has yet to be clear data to validate the physiological and clinical implications of the gut-lung axis in CF, but early evidence has hinted at its relevance. Hoen et al. discovered that in children with CF, pulmonary colonisation with the pathogen *Pseudomonas aeruginosa*, which leads to respiratory failure, was preceded by a significant depletion of *Parabacteroides* in the intestinal microbiome [159]. *Parabacteroides* is a genus associated with immunomodulatory and anti-inflammatory properties, and its reduction prior to pulmonary *P. aeruginosa* colonisation may corroborate the critical role of gut microbes in host responses to threats to the respiratory tract [160,161]. Furthermore, one study reported an association between gut microbial diversity and pulmonary exacerbation events in CF [128], and another found that species diversity was significantly reduced in patients with lower FEV1 compared to those with better lung function [125]. Positive correlations between FEV1 and specific bacterial communities, such as the SCFA-producing *Ruminococcaceae* family, have also been documented [77]. Altogether, these findings suggest that the optimisation of gut health may have profound benefits on pulmonary function, and the gut–lung axis should continue to be explored for future therapeutic considerations.

#### 4.4.2. Growth and Nutritional Status

Nutritional status, as assessed by height and weight, is of paramount importance in CF. Better nutritional status, especially in early life, is associated with better lung function and long-term outcomes [162,163,164,165]. Hayden et al. recently identified that infants with CF who had low length exhibited a more marked dysbiosis than infants with CF who had normal length [115]. In particular, infants with low length had a markedly reduced abundance of Bacteroidetes and a significant increase in Proteobacteria, as well as a further delay in microbiome maturation [115]. A positive correlation between anthropometric measures and certain bacterial genera such as *Ruminococcaceae* has also previously been reported [77]. Additionally, functional data have indicated a decreased propensity of the CF gut microbiota to utilise and synthesise water-soluble vitamins, which typically facilitate nutrient metabolism [77]. Proteomics techniques have also revealed that the CF intestinal microbiome exhibits comparatively fewer proteins known to play a role in carbohydrate transport, metabolism, and conversion [129]. Taken together, these findings demonstrate that the composition and functionality of the gut microbiome are closely intertwined with nutritional status.

## 5. Linking Intestinal Inflammation and Gut Dysbiosis

Given the delicate homeostasis of the enteric environment and the common contributors to both gut inflammation and dysbiosis, a pathophysiological link between these two intestinal sequelae of CF is of interest. The key to unravelling this link may lie in SCFAs, the primary metabolites of the anaerobic fermentation of indigestible dietary fibres by bacteria in the colon [166,167,168,169]. Butyrate, acetate, and propionate are the main SCFAs produced by the gut microbiota and have been the focus of most SCFA-related research, particularly butyrate [170,171,172]. They perform various important functions, including providing nourishment for colonocytes, maintaining the gut epithelium, regulating intestinal pH, and modulating the immune response. Therefore, they are postulated to have important implications in intestinal disease [122,166,170]. In support of this, SCFAs have been shown to improve epithelial integrity and ameliorate intestinal inflammation in numerous animal models [173,174,175,176,177,178]. 

Many of the SCFA-producing organisms belong to the major commensal phyla Firmicutes and Bacteroidetes, such as *F. prausnitzii*, *Roseburia* spp., and *Bacteroides* spp., which are depleted in the CF gut, as discussed above [179]. The correlation between reduced abundances of SCFA producers and intestinal inflammation in CF is strengthened by evidence that levels of butyrate, propionate, and acetate are lower in children with CF compared to healthy controls [77,116]. Furthermore, it has been shown that the CF gut microbiome possesses enriched genes for SCFA catabolism, the number of which positively correlates with fecal calprotectin [77,131]. Many of the same factors that contribute to both intestinal inflammation and dysbiosis, notably, antibiotic use, the high-fat CF diet, and prolonged intestinal transit, are also associated with reduced SCFA levels [180,181,182,183,184,185]. Altogether, there is an increasingly robust body of evidence to demonstrate that gut microbes play a critical role in preventing and ameliorating intestinal inflammation.

However, the relationship is likely also bidirectional, as an inflamed enteric environment confers growth advantage for organisms that are able to withstand the metabolic changes associated with inflammation. During inflammation, reactive oxygen and nitrogen species are produced by inflammatory cells. These reactive species supply terminal electron acceptors required for anaerobic respiration and facilitate the proliferation of organisms with the ability to efficiently perform anaerobic respiration [186]. Intestinal inflammation is strongly associated with the bloom of *Enterobacteriaceae*, a family of bacteria that exhibits very high nitrate reductase activity and can thus utilise nitrate respiration for growth [187,188]. Indeed, a number of organisms belonging to the *Enterobacteriaceae* family, including the aforementioned *Enterobacter* genus and *E. coli* species, are increased in both IBD and CF, corroborating the effect of an inflamed intestine on the selection of microbial communities [44,77,78,111,116,125,127,129,130,189,190,191]. In summary, while the precise mechanisms by which intestinal inflammation and dysbiosis relate to each other are not fully known, evidence of their dynamic relationship reflects a sophisticated enteric environment inundated with complex interactions and functions.

## 6. CF Intestinal Disease in the Era of CFTR Modulator Therapies

The recent years have ushered in a new era in which CFTR modulators have begun to, and will continue to, revolutionise the management of CF. Ivacaftor, one of the earliest approved therapies, is a CFTR potentiator that augments anion transport in patients with gating mutations [192,193]. On the other hand, the Lumacaftor–Ivacaftor combination therapy also includes Lumacaftor, a CFTR corrector for patients with mutations in which the CFTR protein is misprocessed and largely unable to reach the cell surface [194]. It is well-documented that Ivacaftor and Lumacaftor/Ivacaftor are associated with weight gain, although the mechanisms by which this occurs are yet to be fully elucidated [65,66,192,193,194]. The improvement in nutritional status with CFTR modulation therapy is likely of multifactorial origin, and the role of GI-related outcomes is increasingly recognised. Stallings et al. demonstrated that Ivacaftor-induced weight gain was correlated with decreased fecal calprotectin and increased dietary fat absorption in PI patients [66]. Notably, they did not report significant changes in fecal elastase levels, highlighting that improved fat absorption may occur as a result of CFTR modulation directly in the intestinal tract rather than the exocrine pancreas [66]. This suggests that the benefits of Ivacaftor may be more profound in the intestinal tract than in the pancreas. Ivacaftor has also been shown to reduce gastrointestinal pH, possibly by facilitating bicarbonate secretion, which may subsequently reduce intestinal inflammation [192,195]. Additionally, Ivacaftor has been reported to promote the resolution of intestinal histopathological changes (i.e., inspissated mucus in intestinal crypts) typically seen in CF [48]. In light of the aforementioned associations between intestinal inflammation and growth parameters, the attenuation of gut inflammation by CFTR modulators, as evidenced by reductions in fecal calprotectin levels, may confer a potential to improve growth and nutrition [64,65,66]. 

An emerging body of evidence also suggests that modulator therapies could impact the gut microbiota, which may, in turn, alter the course of intestinal inflammation. Ooi et al. reported that reductions in fecal calprotectin following treatment with Ivacaftor were associated with decreased abundances of *Enterobacteriaceae* [65]. Moreover, they observed increased abundances of the anti-inflammatory genus *Akkermansia* in association with normal fecal M2-PK levels after the initiation of Ivacaftor [65]. Recently, Kristensen et al. also reported that Ivacaftor treatment was associated with a significant increase in gut microbial diversity [196]. It is postulated that these shifts in the microbiome after CFTR modulation may be due to the combined selective effects of alterations in ion and fluid balance, dietary changes initiated with Ivacaftor treatment, and reductions in antibiotic use following improvements in respiratory function [65,196].

Given the relative novelty of CFTR modulator therapies, there remain many unknowns, particularly as GI endpoints are not generally included in evaluations of the efficacy of modulator therapies. The few GI-specific studies to date have also been very limited in sample sizes and varied in their study populations. For example, Pope et al. [197] did not observe any significant changes in the gut microbiota following the initiation of Ivacaftor or Lumacaftor/Ivacaftor, contrary to the aforementioned findings by Ooi et al. [65] and Kristensen et al. [196]. However, the study by Pope et al. [197] involved a cohort of all PS patients with an R117H allele, whereas Ooi et al. [65] included predominantly PI patients with a G551D mutation, and Kristensen et al. [196] studied a cohort of primarily PI patients with an S1251N mutation. These differences reflect a potentially high degree of variability in GI responses to modulator therapies contingent on genotype and exocrine pancreatic status. As such, the comparability of the existing studies is lessened. As the use of CFTR modulators becomes more widespread, larger and longer-term studies involving diverse patient cohorts are necessary to strengthen current knowledge of the GI benefits of these drugs.

## 7. Future Directions

### 7.1. Probiotics

Considering the evidence of intestinal dysbiosis in CF and its probable links to intestinal inflammation and overall wellbeing, the therapeutic manipulation of the gut microbiota has garnered interest. Probiotic therapy refers to the supplementation of live microbes that may confer health benefits to the host. Some of the most common probiotic strains in experimental CF cohorts include *Lactobacillus reuteri*, *Lactobacillus rhamnosus GG*, as well as mixed-strain preparations [198]. To date, there has been a very limited number of high-quality clinical trials to validate the utility of probiotics in the general management of CF, but the findings from a small group of studies have revealed positive pulmonary and GI outcomes with few adverse effects [199,200,201,202]. It is notable that the majority of studies with GI endpoints have reported a reduction in fecal calprotectin levels following probiotic treatment in patients with CF. These findings may prompt patients and healthcare providers to consider probiotics [81]. 

In addition to a reduction in fecal calprotectin, probiotics have also been shown to partially restore the CF gut microbiome to a state that is more similar to that of a healthy gut [41]. For example, del Campo et al. [80] reported a reduction in gamma-Proteobacteria, which generally exists in increased relative abundances in CF, following probiotic administration. However, there is marked heterogeneity in studies on probiotics in terms of the strains and dosages administered, duration of treatment and follow up, and study design. Furthermore, the implications of these findings are unclear, as there is no long-term or adequately powered data to sufficiently demonstrate their clinical significance [81]. Hence, large-scale, well-designed trials with longitudinal data are necessary to better characterise the possible benefits of probiotic administration. As the pathogenesis of CF intestinal manifestations is multifactorial, the utility of probiotics also needs to be considered in light of the other pathogenetic factors which may or may not be modifiable. The synergistic effects of the concurrent administration of CFTR modulators and probiotics may be one such avenue to explore. All in all, knowledge regarding the applicability of probiotics in CF remains limited, but the pre-existing studies may foreshadow future paradigm shifts in treatment and management to include probiotics and other methods of intestinal microbial modulation.

### 7.2. The Increased Risk of GI Malignancies

As the life expectancy of people with CF has increased, longitudinal data have revealed an increased risk and earlier emergence of GI malignancies [21,22]. A myriad of factors may synergistically contribute to GI carcinogenesis in the context of CF. These include chronic intestinal inflammation, increased intestinal cell turnover (independent of inflammation), intestinal dysbiosis, the high-fat and high-energy CF diet, prolonged immunosuppressive therapy following lung transplantation, and defects in the intrinsic tumour-suppressive functions of CFTR [7,31,57]. Chronic intestinal inflammation has long been established as a significant risk factor for GI malignancies [203,204,205,206,207]. The mechanisms by which inflammation promotes carcinogenesis are not entirely known, but a major contributor is likely oxidative stress induced by ongoing inflammation. Oxidative stress can cause DNA damage and epigenetically interfere with the expression of regulatory proteins, transcription factors, and signalling molecules that normally suppress tumour development [204,205,208]. Moreover, inflammation has been shown to induce shifts in the gut microbiota to favour the expansion of genotoxic organisms, especially *E. coli* [203]. Notably, *E. coli*, which is relatively more abundant in the CF intestine, is also increased in IBD and colorectal cancer, corroborating the potential compounding effects of inflammation and dysbiosis in carcinogenesis [209,210,211]. *Fusobacterium*, a genus that has been extensively linked to colorectal cancer, is also relatively enriched in the CF gut [77]. Additionally, *Faecalibacterium* and *Roseburia*, both SCFA producers, have been observed in reduced abundances in patients with colorectal cancer, as is in CF [212]. Interestingly, alterations in the intestinal virome and mycobiome (outlined in the following section) have also been associated with colorectal cancer [213,214]. Altogether, large-scale longitudinal studies in CF cohorts are warranted to extensively examine the relationship between intestinal inflammation, dysbiosis, and GI carcinogenesis. Treatment of intestinal inflammation and probiotic therapies may be future avenues through which the risk of GI malignancies is reduced in people with CF.

### 7.3. The Intestinal Virome and Mycobiome

Besides intestinal bacteria, the roles of the other components of the gut microbiome, including viruses and fungi, ought to be considered for a broader perspective on host–microbiota interactions. Bacteriophages are viruses that replicate within bacteria and comprise the core commensal constituents of the enteric virome. It is unclear how bacteriophages are involved in intestinal homeostasis, but they are postulated to modulate the bacterial microbiome and, in doing so, indirectly contribute to the enteric environment [215,216]. Alterations in the intestinal virome have been linked to various conditions, including IBD and colorectal cancer [213,217,218,219]. To date, there has only been one study on the CF intestinal virome, which involved eight pediatric patients. Coffey et al. reported that the CF intestinal virome was significantly different from that of healthy controls in terms of both the viral communities present and their predicted functionality [220]. They also noted a significantly decreased abundance of Faecalibacterium phage FP Taranis, a bacteriophage hosted by the anti-inflammatory bacterial species *F. prausnitzii*, in the CF intestine. Furthermore, they observed relatively increased synthesis of bacterial endolysins and increased abundances of bacteriophages associated with Proteobacteria, a bacterial phylum encompassing pathogens such as *Enterobacteria* and *Escherichia*. These factors may all synergistically contribute to enteric inflammation. Notably, Coffey et al. found that certain viral communities were correlated with nutritional status and fecal inflammatory markers [220]. Taken together, the findings from this pilot study highlight that the intestinal virome may play a significant role in mediating intestinal inflammation and nutritional outcomes in CF, thereby warranting further investigations.

The human gut fungal microbiome, referred to as the mycobiome, is an emerging field of research. While knowledge remains relatively sparse, it has been established that the predominant phyla of the healthy gut mycobiome are Ascomycota and Basiodiomycota. Compared to the bacterial microbiome, the mycobiome exhibits less diversity and greater population-wide variability, instability, and susceptibility to environmental factors [221,222,223]. The mechanistic aspects of commensal intestinal fungal colonisation remain largely unknown, but it is hypothesised that fungi may be involved in innate and adaptive immune pathways that confer protective benefits to the host intestinal epithelium [221,224]. Fungal dysbiosis has been associated with some conditions, including IBD, alcoholic liver disease, pancreatic ductal adenocarcinoma, and obesity, but its specific implications in CF have not been explored [225,226,227,228,229,230,231].

### 7.4. Multi-Omics Research

The new phase of research on the CF gut microbiota includes a multi-omics approach involving metagenomics, metatranscriptomics, metaproteomics, and metabolomics. A multi-omics approach can provide sophisticated findings that combine microbial composition, diversity, function, and activity to yield powerful insights into genotype–phenotype and host–microbe correlations [232]. In particular, metaproteomics, the profiling of microbial-associated proteins, is a promising sphere that could alter the future of patient assessment and management in CF. Through metaproteomics, the proteins identified to be associated with clinical features such as inflammation could serve as potential biomarkers of disease and measure responses to treatment [129]. However, there remain several limitations to multi-omics approaches, highlighting the need for the active development of these techniques. There is currently no standardised protocol for the extraction of proteins from fecal samples or computational analysis of data obtained through metaproteomics methods [233]. Fecal samples may also be prone to processing errors and contamination, particularly during freezing and thawing, which could significantly impact the recovery of key proteins and metabolites. Additionally, owing to the relative novelty of multi-omics techniques, robust sequence databases are still lacking, and a high level of analytical computing is required to interpret data, posing a technical challenge [122,232]. Notwithstanding these limitations, continued research utilising a multi-omics approach can propel current understanding of the CF intestinal microbiome and its functionality to greater heights and elucidate findings that can potentially shape the next chapter of CF treatment and management.

## 8. Conclusions

Intestinal inflammation and alterations in the gut microbiota arise from a multitude of intrinsic and extrinsic factors and are associated with important clinical outcomes in CF. The interrelatedness of these two enteric phenomena highlights the complexity and sophistication of the intestinal milieu. Additionally, the emergence of evidence linking gut inflammation and microbial dysbiosis with clinical measures emphasises the clinical relevance of these manifestations. With the rise of new treatments and advanced technological modalities, the gastrointestinal manifestations of CF ought to be research priorities. Further investigations of the CF intestine may reveal pivotal insights that could yield substantial long-term benefits for people with CF.

## Data Availability

Not applicable.
